# Biological Attachment Systems and Biomimetics—In Memory of William Jon P. Barnes

**DOI:** 10.3390/biomimetics10040220

**Published:** 2025-04-02

**Authors:** Thies H. Büscher, Stanislav N. Gorb

**Affiliations:** Functional Morphology and Biomechanics, Zoological Institute, Kiel University, D-24118 Kiel, Germany; sgorb@zoologie.uni-kiel.de

## 1. Introduction to the Special Issue

Any system preventing the separation of two surfaces may be defined as an attachment system. Such systems are common in nature, aiding in locomotion [1], settlement [2], mating [3], and many more functions. These biological attachment systems (BASs) are used to either temporarily or permanently attach an organism to substrates, to other organisms, or for the temporary interconnection of body parts within an organism [4]. For this, BASs can either employ entirely mechanical principles, or additionally, rely on surface chemistry and incorporate fluids in the contact region. The structure and physical mechanisms of BASs vary enormously and are subject to different functional loads, due to their specific areas of application. Because of this, many functional solutions have evolved independently in different lineages of organisms [5]. Many species of animals and plants exhibit diverse BASs that differ in their morphology depending on the biology of the species and the particular function in which the corresponding BAS is involved [6]. However, all BASs rely on similar physical and chemical principles. This connection between specific problem solving and the usage of general physical principles renders BASs a promising field of research for biomimetics.

This Special Issue provides recent insights into state-of-the-art basic research on BASs and derived biomimetic studies. It showcases the width of research in the field of attachment systems across biological taxa and disciplines. We appreciate the diversity of contributions to this Special Issue and would like to thank the colleagues that kindly accepted our invitation. Their dedication enabled this collection of articles on biological attachment phenomena from a wide range of perspectives. The published articles cover topics from a range of biological taxa to experimental studies of their adhesive mechanisms, including *Dictyostelium* cells [7], cnidarians [8], molluscs [9], insects [10,11,12] and plant seeds [13]. Because of the diversity of functions in BASs, biology could provide interesting inspirations for the design and fabrication of biomimetic attachment devices. Furthermore, biological studies are complemented with experiments and simulations investigating the properties of artificial materials involved in adhesive contact formation [14] and interfacial mechanisms [15]. The combination of such original biological, physical, and engineering studies is the foundation for biomimetic innovation, as demonstrated in contributions on mushroom-shaped biomimetic microstructures in this Special Issue [16].

The experimental insights in this collection are accompanied by two review articles from both perspectives, elaborating on the methodological considerations for experiments on biological systems [17] and the structure–function relationships of friction control in bioinspired systems [18]. In summary, the reader can expect a wide overview of attachment-related phenomena, spanning from insights into the mechanisms of diverse taxa to bioinspired engineering.

## 2. Dedication

**Figure 1:**
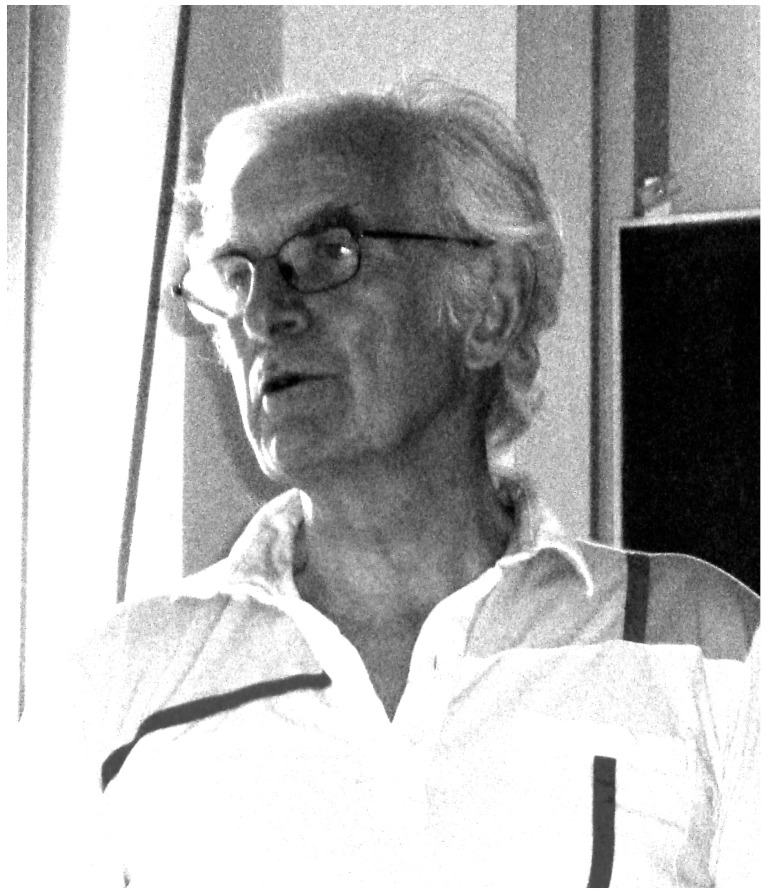
Jon Barnes in August 2011, giving a lecture at Kiel University, Germany.

This Special Issue is dedicated to Prof. Dr. Jon Barnes. With sadness, we took note that William Jon Peter Barnes suddenly passed on 16 April 2024 in his home in Glasgow at the age of 83 years. Jon Barnes (Figure 1) was a well-known and enthusiastic specialist in the field of biological adhesion. He published various articles that were important milestones for our understanding of adhesion and contact phenomena in biological systems.

In his research, Jon Barnes combined various morphological and experimental approaches, field studies, and modern microscopical and force measurement techniques that significantly promoted knowledge in the field of biological adhesion. His work was dedicated to the understanding of tree frog adhesion, and contributed to the in-depth understanding of this particular adhesive system. His contributions gave rise to various applied aspects that were integrated in large-scale industrial developments, such as the development of bioinspired profiles for winter tires [19,20]. In addition to the major impact of his research on the community, Jon Barnes left significant marks in the fields of neuroethology, animal physiology, and behavior.

Jon Barnes was well respected in the adhesion community, and his elegant combination of high-quality basic research and applied aspects were highly influential for many scientists. As a leading scientist with an established reputation worldwide, he passed his knowledge onto colleagues in various conferences and by organizing several symposia on his related research topics. In this manner, his achievements were acknowledged in the symposium ‘*Biomechanics of arboreal locomotion—a tribute to Jon Barnes*’, organized by Walter Federle at the 2007 annual meeting of the Society for Experimental Biology in Glasgow, where Jon Barnes resided. At the University of Glasgow, he was a valued colleague and teacher who received excellent assessments from his students over the years and demonstrated a strong ability to influence, motivate, and inspire his students, and from October 2006, he carried on his commitment as Honorary Research Fellow. As well as his position in Glasgow, Jon Barnes spent several Alexander von Humboldt Fellowships at various universities over the years, such as the universities of Konstanz (1974/5), Frankfurt (1986), and Würzburg (2005). Alongside his academic passion, he dedicated himself to local nature conservation with the Scottish Wildlife Trust over the decades. He was actively involved with this organization from the late 1960s, and served as member of the Trust’s Council and Convenor of the Conservation Committee for decades, including periods as Vice-Chairman (1985 to 2003) and Chairman (2003 to 2006).

His dedication to research in the field of bioadhesion and his ambitious contributions to nature conservation will truly be missed. Jon Barnes actively devoted himself to the field and continued to pursue his fascination with tree frog adhesive systems far beyond his retirement. During the compilation of this Special Issue, he was actively working on another manuscript on the influence of surface energy on tree frog attachment, until his passing stopped him from finishing his work.

## References

[1] Büscher T.H., Gorb S.N. (2021). Physical constraints lead to parallel evolution of micro-and nanostructures of ani-mal adhesive pads: A review. Beilstein J. Nanotechnol..

[2] Aldred N., Clare A.S., Gorb S.N. (2009). Mechanisms and principles underlying temporary adhesion, surface exploration and settlement site selection by barnacle cyprids: A short review. Functional Surfaces in Biology.

[3] Miller P.L. (1991). The structure and function of the genitalia in the Libellulidae (Odonata). Zool. J. Linn. Soc..

[4] Gorb S.N. (2001). Attachment Devices of Insect Cuticle.

[5] Büscher T.H., Gorb S.N., Bels V.L., Russell A.P. (2023). Convergent Evolution of Animal Adhesive Pads. Convergent Evolution. Fascinating Life Sciences.

[6] Spolenak R., Gorb S.N., Arzt E. (2005). Adhesion design maps for bio-inspired attachment systems. Acta Biomater..

[7] Fakhari S., Belleannée C., Charrette S.J., Greener J. (2024). A microfluidic design for quantitative measurements of shear stress-dependent adhesion and motion of *Dictyostelium discoideum* cells. Biomimetics.

[8] Seabra S., Zenleser T., Grosbusch A.L., Hobmayer B., Lengerer B. (2022). The involvement of cell-type-specific glycans in *Hydra* temporary adhesion revealed by a lectin screen. Biomimetics.

[9] Xi P., Qiao Y., Cong Q., Cui Q. (2024). Experimental study on the adhesion of Abalone to surfaces with different morphologies. Biomimetics.

[10] Thomas J., Gorb S.N., Büscher T.H. (2023). Characterization of morphologically distinct components in the tarsal secretion of *Medauroidea extradentata* (Phasmatodea) using cryo-scanning electron microscopy. Biomimetics.

[11] Grohmann C., Cohrs A.-L., Gorb S.N. (2022). Underwater attachment of the water-lily leaf beetle *Galerucella nymphaeae* (Coleoptera, Chrysomelidae). Biomimetics.

[12] Goetzke H.H., Burrows M., Federle W. (2025). Mantises Jump from Smooth Surfaces by Pushing with “Heel” Pads of Their Hind Legs. Biomimetics.

[13] Büscher T.H., Gorb S.N. (2022). Convergent evolution of adhesive properties in leaf insect eggs and plant seeds: Cross-kingdom bioinspiration. Biomimetics.

[14] Lyashenko I.A., Popov V.L., Borysiuk V. (2023). Indentation and detachment in adhesive contacts between soft elastomer and rigid indenter at simultaneous motion in normal and tangential direction: Experiments and simulations. Biomimetics.

[15] Lin Z., Xiao K., Li L., Zhang Y., Zhang X., Chen D., Xue L. (2023). The influence of temperature on anisotropic wettability revealed by friction force measurement. Biomimetics.

[16] Gonen M., Kasem H. (2023). Effect of the mechanical properties of soft counter-faces on the adhesive capacity of mushroom-shaped biomimetic microstructures. Biomimetics.

[17] van den Boogaart L.M., Langowski J.K.A., Amador G.J. (2022). Studying stickiness: Methods, trade-offs, and perspectives in measuring reversible biological adhesion and friction. Biomimetics.

[18] Kong Y., Ma S., Zhou F. (2024). Bioinspired interfacial friction control: From chemistry to structures to mechanics. Biomimetics.

[19] Kappl M., Kaveh F., Barnes W.J.P. (2016). Nanoscalefriction and adhesion of tree frog toe pads. Bioinspir. Biomim..

[20] Iturri J., Xue L., Kappl M., García-Fernández L., Barnes W.J.P., Butt H.-J., del Campo A. (2015). Torrent frog-inspired adhesives: Attachment to flooded surfaces. Adv. Funct. Mater..

